# LGR6 is a potential diagnostic and prognostic marker for esophageal squamous cell carcinoma

**DOI:** 10.1002/jcla.23121

**Published:** 2020-01-09

**Authors:** Tianci Chai, Zhimin Shen, Zhenyang Zhang, Sui Chen, Lei Gao, Peipei Zhang, Wenwei Lin, Mingqiang Kang, Jiangbo Lin

**Affiliations:** ^1^ Department of Thoracic Surgery Fujian Medical University Union Hospital Fuzhou China; ^2^ Department of anesthesiology Xinyi People's Hospital Xuzhou China; ^3^ Key Laboratory of Ministry of Education for Gastrointestinal Cancer Fujian Medical University Fuzhou China

**Keywords:** esophageal squamous cell carcinoma, immunohistochemistry, LGR6, prognosis

## Abstract

**Background:**

Leucine‐rich repeat–coupled receptor 6 (LGR6) is a marker of the skin, nails, and other types of adult tissue stem cells and has been widely found to be related to the development and progression of a variety of cancer types. The clinical significance and biological function of LGR6 in esophageal squamous cell carcinoma (ESCC) have not been determined.

**Methods:**

The expression of LGR6 at the transcriptional level was analyzed by searching the TCGA and UCSC data sets. Immunohistochemistry, WB, and q‐PCR were used to detect the expression of LGR6 in ESCC and adjacent normal tissues. LGR6 PPI networks and KEGG pathways were used to analyze the potential biological functions of LGR6.

**Results:**

The expression of LGR6 in ESCC tissues was significantly higher than that in normal tissues and was negatively correlated with the differentiation degree of ESCC and the prognosis of the patients but not closely correlated with the TNM stage of ESCC. PPI networks showed that LGR6 had a close interaction with RSPO1, RSPO2, RSPO3, and RSPO4. KEGG pathway analysis showed that LGR6 activated the Wnt/β‐catenin signaling pathway by binding with RSPO ligands to promote the progression of ESCC.

**Conclusion:**

LGR6 can serve as a potential diagnostic and prognostic marker for ESCC.

**Abbreviations**
AJCC: American Joint Committee on Cancer
ESCC: esophageal squamous cell carcinoma
KEGG: Kyoto encyclopedia of genes and genomes
LGR6: leucine‐rich‐repeat‐coupled receptor 6
PPI: protein‐protein interaction
STRING: search tool for the retrieval of interacting genes
TCGA: the cancer genome atlas
UCSC: University of California Santa Cruz
WB: Western blot

## 1 INTRODUCTION

Esophageal cancer is one of the most common malignant tumors in the world, and its incidence ranks seventh among those of all malignant tumors.^1^ Esophageal cancer can be divided into two pathological types: squamous cell carcinoma (SCC) and adenocarcinoma. Esophageal squamous cell carcinoma (ESCC) is the most common type of esophageal cancer in Asian countries, accounting for approximately 80% of all esophageal cancers.^2^ Gastroscopy is an effective diagnostic method for esophageal cancer, but most of the patients are in an advanced stage at diagnosis. Even with active surgery and neoadjuvant therapy, the prognosis is still poor. The identification of new, reliable diagnostic, and targeted therapeutic molecular biomarkers associated with the clinical factors of esophageal cancer is critical.^3^

Leucine‐rich repeat‐containing G protein–coupled receptors (LGRs) are a subgroup of the seven‐transmembrane G protein‐coupled superfamily that regulates various physiological processes associated with various diseases,^4^ and its member LGR4‐6 has high homology.^5^ Many studies have recently explored the biological functions of LGR4‐6 in various human cancer types.^6^, ^7^, ^8^, ^9^, ^10^, ^11^, ^12^ LGR4‐6 plays an important role in activating the Wnt/β‐catenin pathway by binding with R‐spondin (RSPO) ligands, which are closely related to tumor progression and invasion.^10^, ^11^, ^12^

LGR6 plays a pivotal role in adult stem cells, which are markers of various types of adult stem cells in the skin, nails, and a group of basal and intraluminal progenitors that induce luminal tumorigenesis.^13^, ^14^ LGR6 can promote the self‐renewal and progression of non–small‐cell lung cancer and has strong carcinogenic potential.^15^ LGR6 is highly expressed in gastric and colon cancer tissues and is associated with local tumor growth.^16^, ^17^ Recent studies have shown that LGR6 enhances stemness and chemoresistance in ovarian cancer cells by activating the Wnt/β‐catenin signaling pathway.^18^ However, to date, there has been no research on the correlation between the expression of LGR6 at the protein level and clinical‐pathological factors and prognostic features in patients with ESCC.

In this study, we evaluated the clinical significance of LGR6 expression in 102 patients with ESCC and analyzed its association with clinicopathological features and patient survival. We explored the functional effects and potential molecular mechanisms of LGR6 on ESCC progression by using the University of California Santa Cruz (UCSC) and the Cancer Genome Atlas (TCGA) data, constructing a protein‐protein interaction (PPI) network and performing Kyoto Encyclopedia of Genes and Genomes (KEGG) pathway analysis.

## 2 MATERIALS AND METHODS

### 2.1 Clinical samples

During the surgical resection of esophageal cancer at Fujian Medical University Union Hospital (Fuzhou, China) from January 2009 to December 2011, ESCC samples and their corresponding non‐tumor esophageal tissues were collected (n = 102). Tissues were immediately frozen in liquid nitrogen and stored in a −80°C freezer or fixed in 10% formalin for paraffin embedding. All samples were collected with informed consent of the patients, and the study was approved by the institutional review committee and regulatory authorities of Fujian Medical University.

The pathological diagnosis was based on the World Health Organization (WHO) classification, and the clinical‐pathological staging was based on the American Joint Committee on Cancer (AJCC) classification. No patient received chemotherapy or radiation before surgery. The median follow‐up time for overall survival was 43.5 months (range 1‐60 months). The characteristics of the 102 patients with ESCC are summarized in Table 1. The raw clinical data of the patients are shown in Table S1.

**Table 1:** Association between the expression of LGR6 and clinicopathological factors (n = 102)

Characteristics	Value/number of patients	Low expression no (%)	High expression no (%)	*P* value
Age median (range), year	64 (47‐79)			
Gender				
Male	61	26 (42.62)	35 (57.38)	.183
Female	41	13 (31.71)	28 (68.29)	
Age, year				
<60	33	15 (45.45)	18 (54.55)	.181
≧60	69	24 (34.78)	45 (65.22)	
Tumor nodes Metastases category				
T1‐T2	35	14 (40.00)	21 (60.00)	.478
T3‐T4	67	25 (37.31)	42 (62.69)	
N0	55	21 (38.18)	34 (61.82)	.576
N1‐N3	47	18 (38.30)	29 (61.70)	
M0	102	39 (38.24)	63 (61.76)	/
M1	0	0	0	
American Joint Committee on Cancer category				
Ⅰ	18	10 (55.56)	8 (44.44)	.112
Ⅱ	40	11 (27.50)	29 (72.50)	
Ⅲ	44	18 (40.91)	26 (59.09)	
Differentiation				
High	41	26 (63.41)	15 (36.59)	**<.001** *
Middle	39	10 (25.64)	29 (74.36)	
Low	22	3 (13.64)	19(86.36)	
5‐y survival				
Yes	40	24 (60.00)	16 (40.00)	**<.001** *
No	62	15 (24.19)	47(75.81)	

* Bold values indicate statistical significance with *P* < .05.

### 2.2 RNA extraction, reverse transcription, and real‐time quantitative PCR

Total RNA was extracted from frozen tissue using TRIzol reagent (Ambion) according to the manufacturer's instructions, and 1 mg of RNA was reverse transcribed for first complementary DNA strand synthesis using a miScript Reverse Transcription Kit (Qiagen). Real‐time quantitative PCR was performed using a SYBR Premix EX Taq Kit (Takara). The relative mRNA expression of LGR6 was detected with the 2−ΔΔct method using specific primers, and its expression level was normalized to that of endogenous β‐actin. All primers were designed by BioSune Biotechnology Co., Ltd. The sequences of the primers used are as follows: LGR6: 5′‐TGGGGAACCCTCTGCTACAG‐3′ (forward) and 5′‐CAGGTACTGGAATGCCGATCT‐3′ (reverse); and β‐actin: 5′‐CTCCATCCTGGCCTCGCTGT‐3′ (forward) and 5′‐GCTGTCACCTTCACCGTTCC‐3′ (reverse).

### 2.3 Western blot analysis

Tissues were lysed in Western and radioimmunoprecipitation assay (RIPA) tissue lysis buffer (Beyotime) supplemented with phenylmethylsulfonyl fluoride (PMSF; Amresco) on ice for 30 minutes and then centrifuged at 12 000 rpm at 4°C for 10 minutes. The supernatant was collected as the total protein, and then, the protein concentration was determined using a BCA Protein Assay Kit (Thermo Scientific). The same amount (60 μg) of protein in each well was separated by 10% sodium dodecyl sulphate‐polyacrylamide gel electrophoresis (SDS‐PAGE) and transferred to a 0.45‐μm polyvinylidene fluoride (PVDF) membrane (Amersham Hybond, GE Healthcare). The PVDF membrane was then blocked with 0.5% bovine serum albumin (Amresco) followed by incubation overnight at 4°C with primary antibodies against LGR6 (1:2000, Abcam) and β‐actin (1:2000, Abcam). The PVDF membrane was then washed three times for 10 minutes each in Tris‐buffered saline Tween (TBST) at room temperature and then incubated with the secondary antibody for 1 hour at room temperature. The protein imprint was developed by enhanced chemiluminescence (Lulong Biotech).

### 2.4 Immunohistochemistry (IHC)

ESCC and adjacent normal tissues from 102 patients were fixed with formalin and embedded in paraffin, followed by IHC studies using a human anti‐LGR6 antibody (1:200, Abcam). The degree of LGR6 staining was calculated and quantified according to the following aspects: score of stained tumor cells (0, ≤5% positive stained cells; 1, 5%‐25% positive stained cells; 2, 26%‐50% positive stained cells; 3, 51%‐75% positive stained cells; and 4, ≤75% positive stained cells) multiplied by the staining intensity score (0, no staining; 1, weak staining, light yellow; 2, moderate staining, yellowish brown; and 3, strong dye, brown) to obtain the final score. A final score of 3 or lower was classified as low expression, and a final score of 4‐12 points was classified as high expression. The above scoring process was completed by two pathologists (Xinjian Lin and Wannan Chen) in an independent blinded manner. Before an agreement was reached, the two pathologists discussed any inconsistent scores.

### 2.5 LGR6 RNA‐seq analysis based on UCSC and TCGA data

Differences in the mRNA expression of LGR6 in different normal tissues, as well as in different cancer tissues and the corresponding adjacent normal tissues, were investigated by searching the UCSC (http://genome.ucsc.edu/) and TCGA (http://cancergenome.nih.gov) databases.

### 2.6 PPI network construction

Using the Search Tool for the Retrieval of Interacting Genes (STRING) database (http://www.string-db.org/), a PPI network related to LGR6 was established. The interactions procured included known interactions and predicted interactions.

### 2.7 KEGG pathway analysis

The signaling pathways involving LGR6 were determined with the KEGG pathway database (https://www.kegg.jp/kegg/pathway.html), and the function of the LGR6 gene was analyzed.

### 2.8 Statistical analysis

Statistical analysis was performed using SPSS 24.0 for Windows (SPSS, Inc). Student's *t* test was used to test the significance of the differences between each of the two groups. All data are expressed as the mean ± standard deviation (SD) of three independent assays. The association between LGR6 expression and the clinicopathological features of ESCC patients was analyzed using the Pearson chi‐square test. The survival curve was plotted using the Kaplan‐Meier method. When *P* < .05, the difference was considered to be significant.

## 3 RESULTS

### 3.1 LGR6 expression is significantly higher in ESCC tissues than in normal tissues

**Figure 1:**
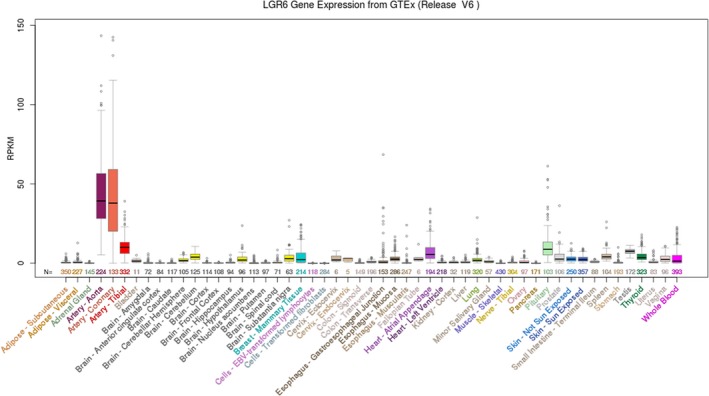
Gene expression in 53 tissues from GTEx RNA‐seq of 8555 samples (570 donors; LGR6). Homo sapiens leucine‐rich repeat containing G protein–coupled receptor 6 (LGR6), transcript variant 2, mRNA (from RefSeq NM_021636). LGR6 mRNA expression in the artery‐aorta median is the highest, and its expression in esophageal tissue is relatively low

**Figure 2:**
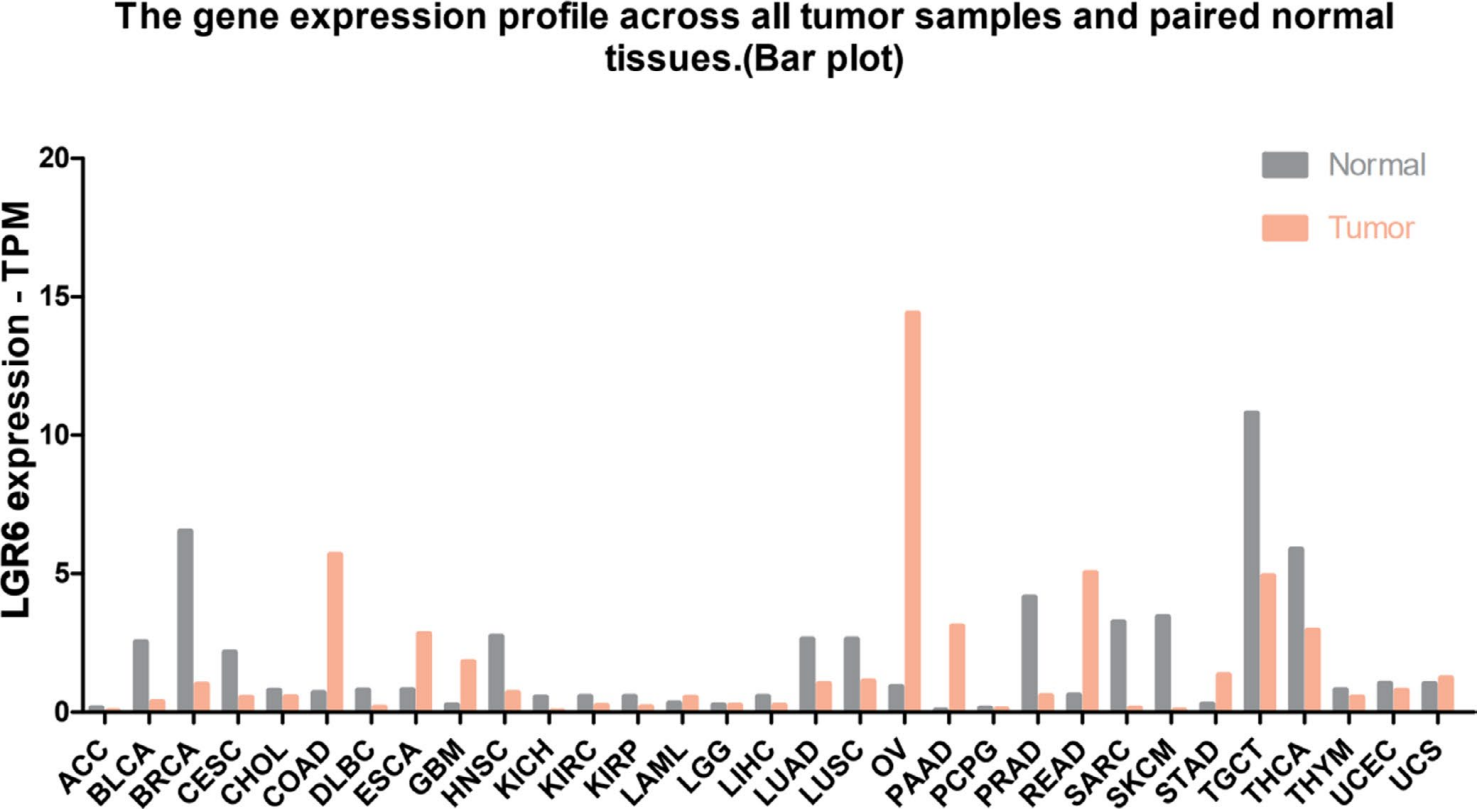
Gene expression profile across all tumor samples and paired normal tissues (Bar plot). The height of the bar represents the median expression of certain tumor types or normal tissue. LGR6 mRNA expression in colon adenocarcinoma (COAD; N: n = 349, T: n = 275), esophageal carcinoma (ESCA; N: n = 286, T: n = 181), glioblastoma multiforme (GBM; N: n = 207, T: n = 163), ovarian serous cystadenocarcinoma (OV; N: n = 88, T: n = 426), pancreatic adenocarcinoma (PAAD; N: n = 171, T: n = 179), rectum adenocarcinoma (READ; N: n = 318, T: n = 92), and stomach adenocarcinoma (STAD; N: n = 211, T: n = 408) is significantly higher than in the corresponding normal tissue

**Figure 3:**
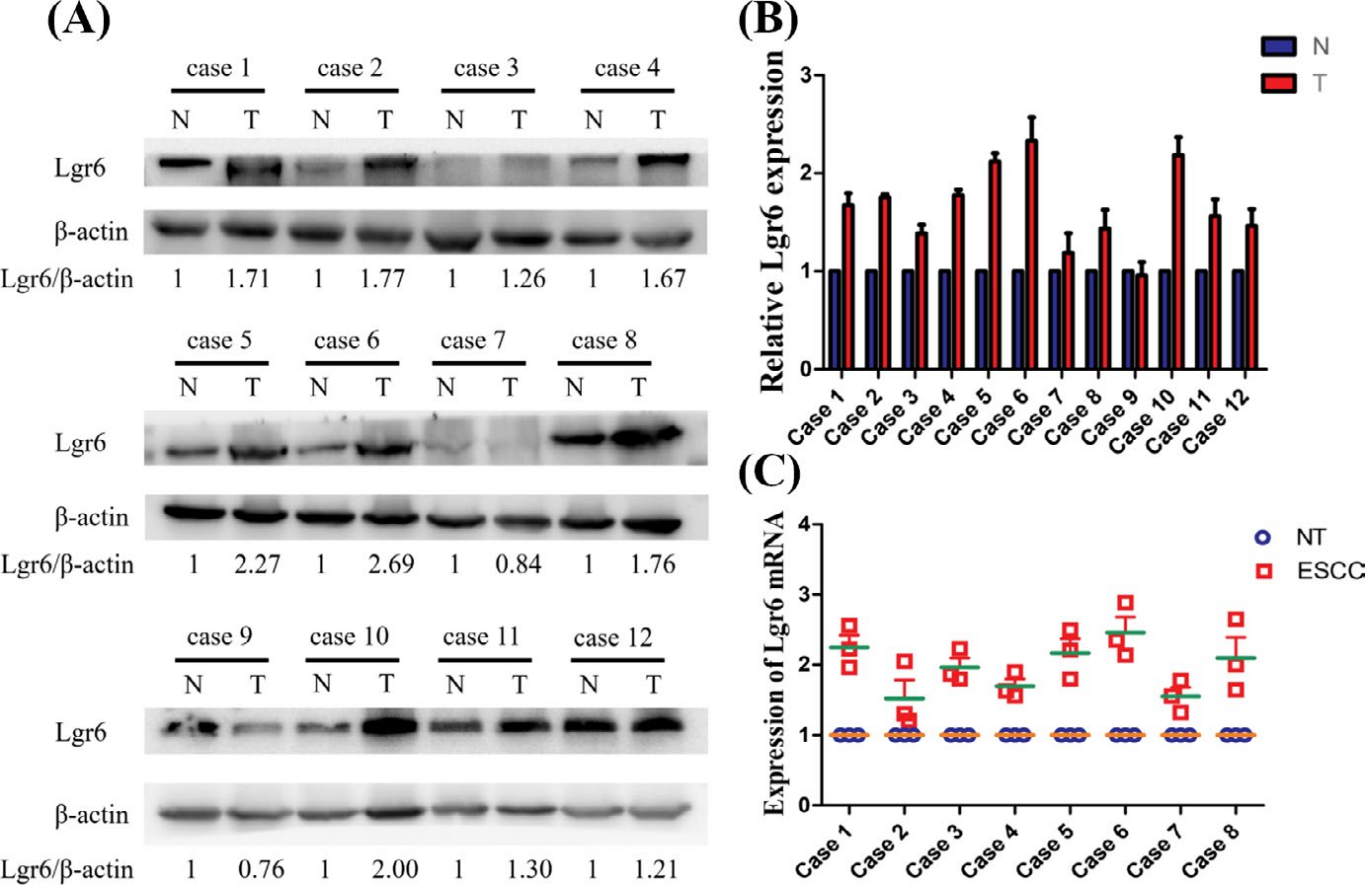
LGR6 expression in ESCC was significantly higher than that in the corresponding non‐cancerous mucosal tissues. Western blot analysis (A and B) and real‐time qPCR (C) results showing the protein and mRNA levels of LGR6 in ESCC tissues and the corresponding non‐cancerous mucosal tissues from twelve randomly chosen patients. β‐Actin serves as the loading control. N and NT represent non‐cancerous normal tissue, and T represents tumor tissue

First, we searched the UCSC data set. Among the 53 tissues obtained from 8555 samples (570 donors), LGR6 mRNA expression was highest in the artery‐aorta, and its expression in esophageal tissue was relatively low (Figure 1). Next, we studied the expression differences in LGR6 mRNA in 31 human cancers compared with normal tissues based on the TCGA and UCSC data sets and found that LGR6 mRNA expression was significantly higher in colon adenocarcinoma (COAD), esophageal carcinoma (ESCA), glioblastoma multiforme (GBM), ovarian serous cystadenocarcinoma (OV), pancreatic adenocarcinoma (PAAD), rectum adenocarcinoma (READ), and stomach adenocarcinoma (STAD) tissues than in the corresponding normal tissues (Figure 2). The above finding was based on the UCSC and TCGA data sets using high‐ or low‐throughput sequencing as a means of studying the expression of LGR6. Western blot analysis and qRT‐PCR confirmed the increased LGR6 protein and mRNA levels in esophageal cancer tissues compared with adjacent normal tissues (Figure 3).

**Figure 4:**
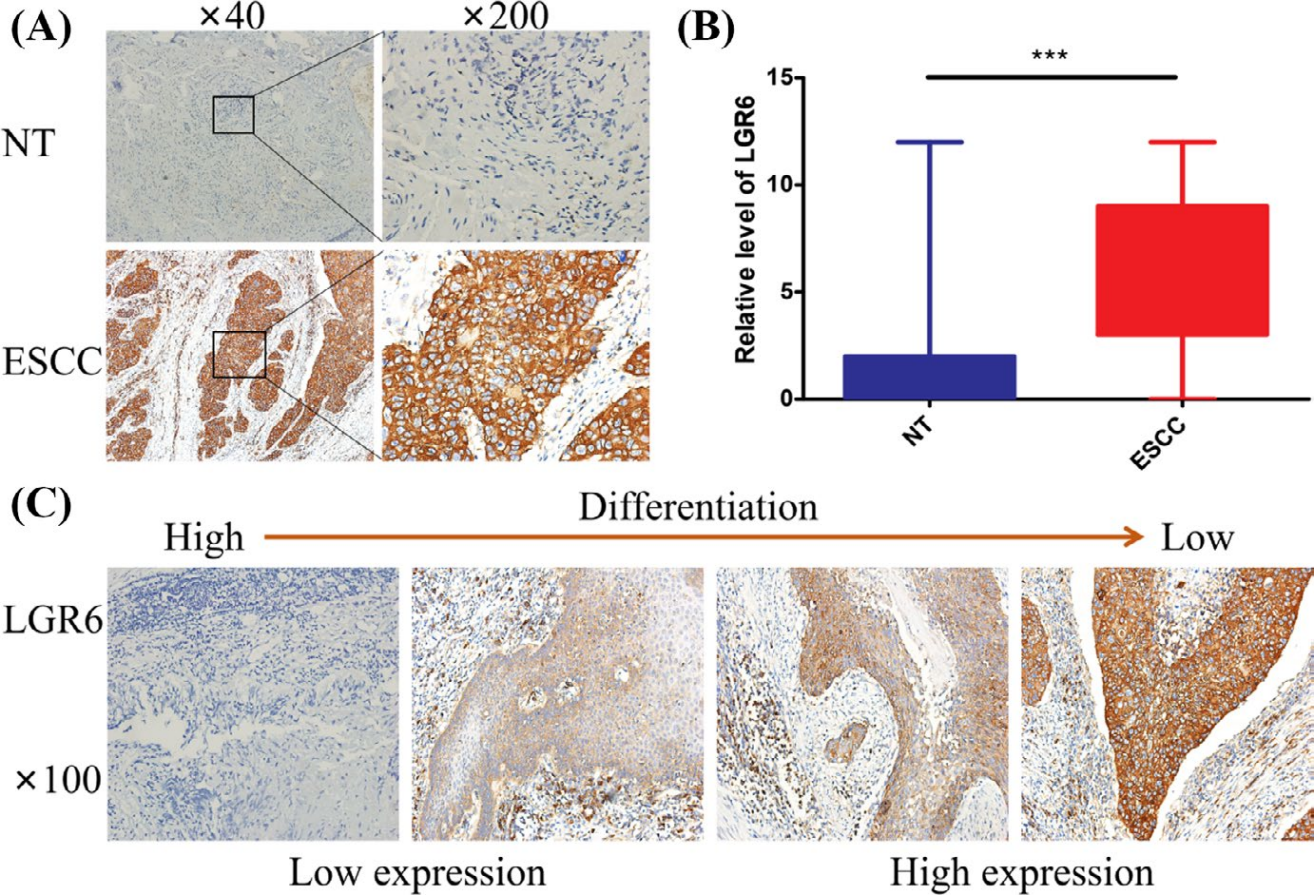
Expression of LGR6 in ESCC and normal esophageal tissues. (A) NT represents normal esophageal mucosal tissue, ESCC represents esophageal squamous cell carcinoma; (B) LGR6 expression in ESCC is higher than in the adjacent normal esophageal mucosal tissue (paired *T*‐test, *P* < .001); (C) IHC showing the relationship between the degree of differentiation of ESCC and the corresponding expression of LGR6

We used IHC to study LGR6 expression in ESCC tissues and the corresponding adjacent normal tissues (NT) in 102 patients. Representative staining images of LGR6 expression are shown in Figure 4A. Positive immunoreactivity in ESCC was mainly localized in the cell membrane, cytoplasm and extracellular matrix, and the cell membrane, cytoplasm and extracellular matrix were rarely stained in normal tissues. The expression of LGR6 was significantly higher in ESCC tissue than its corresponding adjacent normal tissue (paired *t* test *P* < .001; Figure 4B; Table 2). The lower the differentiation level of the ESCC was, the higher the expression of LGR6 was (Figure 4C).

**Table 2:** LGR6 expression in 102 pairs of ESCC samples and their corresponding adjacent normal mucosal tissue

Tissues	Number of patients	LGR6		*P* value
		Low expression	High expression	
ESCC	102	39 (38.24)	63 (61.76)	**<.001** *
NT	102	97 (95.10)	5 (4.90)	

* Bold values indicate statistical significance with *P* < .05.

### 3.2 LGR6 expression is associated with ESCC patient survival and prognosis

**Figure 5:**
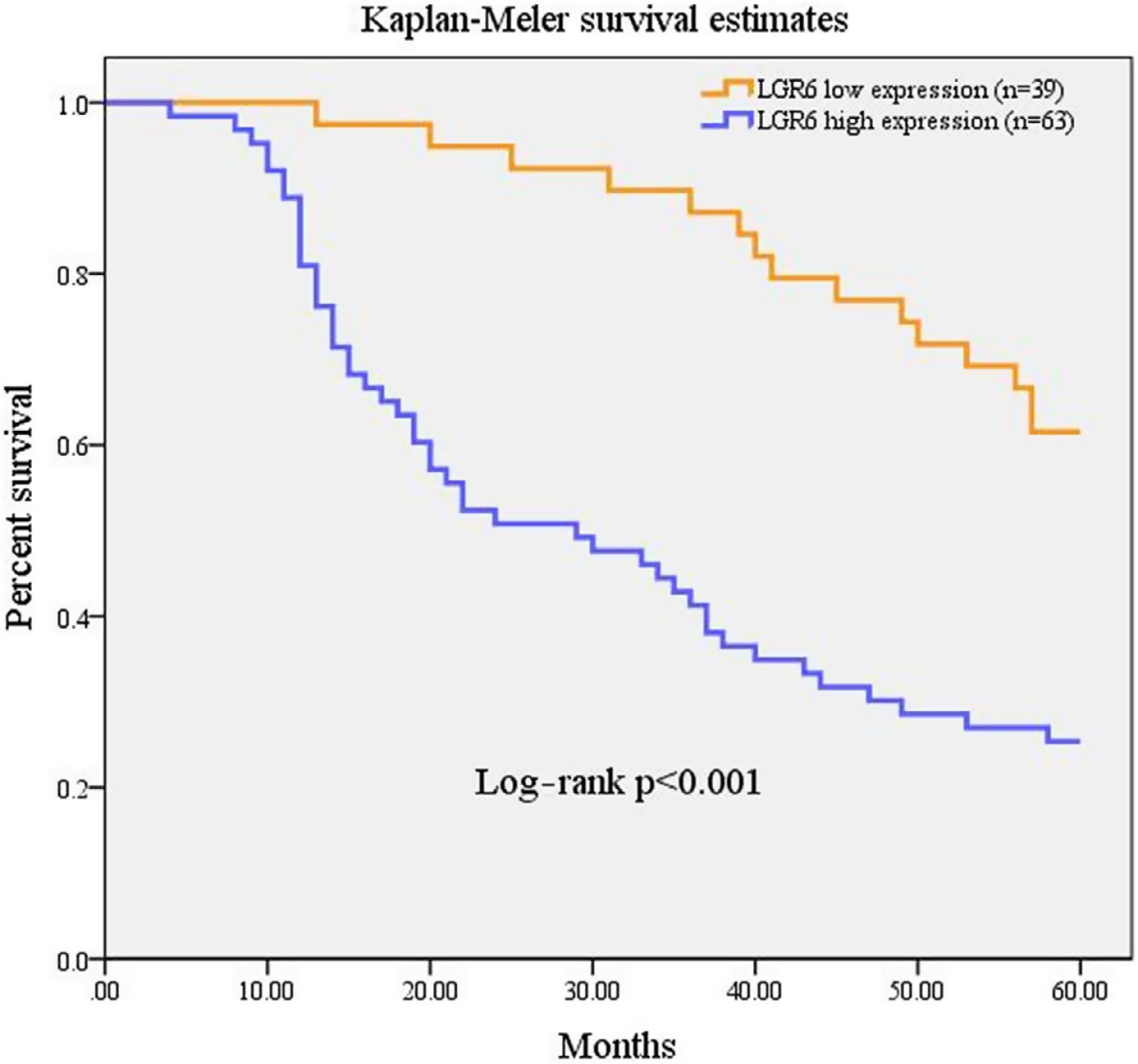
Low expression of LGR6 indicates a longer overall survival of patients with ESCC cancer. Kaplan‐Meier analysis was performed on ESCC patients with high and low expression of LGR6 by the log‐rank test (*P* < .001), and the results show that low expression of LGR6 was correlated with better overall survival of ESCC patients

We analyzed the association between LGR6 expression and clinical features in 102 ESCC patients. Thirty‐nine (38.24%) patients showed low LGR6 expression, and 63 (61.76%) showed high LGR6 expression. LGR6 expression had no obvious correlation with clinical‐pathological variables, including sex (*P* = .183), age (*P* = .181), tumor node metastasis category (T, *P* = .478; N, *P* = .576), or clinical stage (*P* = .112). LGR6 expression was significantly negatively correlated with 5‐year survival (*P* < .001) and differentiation status (Table 1). Kaplan‐Meier survival analysis showed that the 5‐year survival of ESCC patients with high LGR6 expression was significantly lower than that of ESCC patients with low LGR6 expression (log‐rank test, *P* < .001; Figure 5).

### 3.3 PPI network and KEGG pathway construction

**Figure 6:**
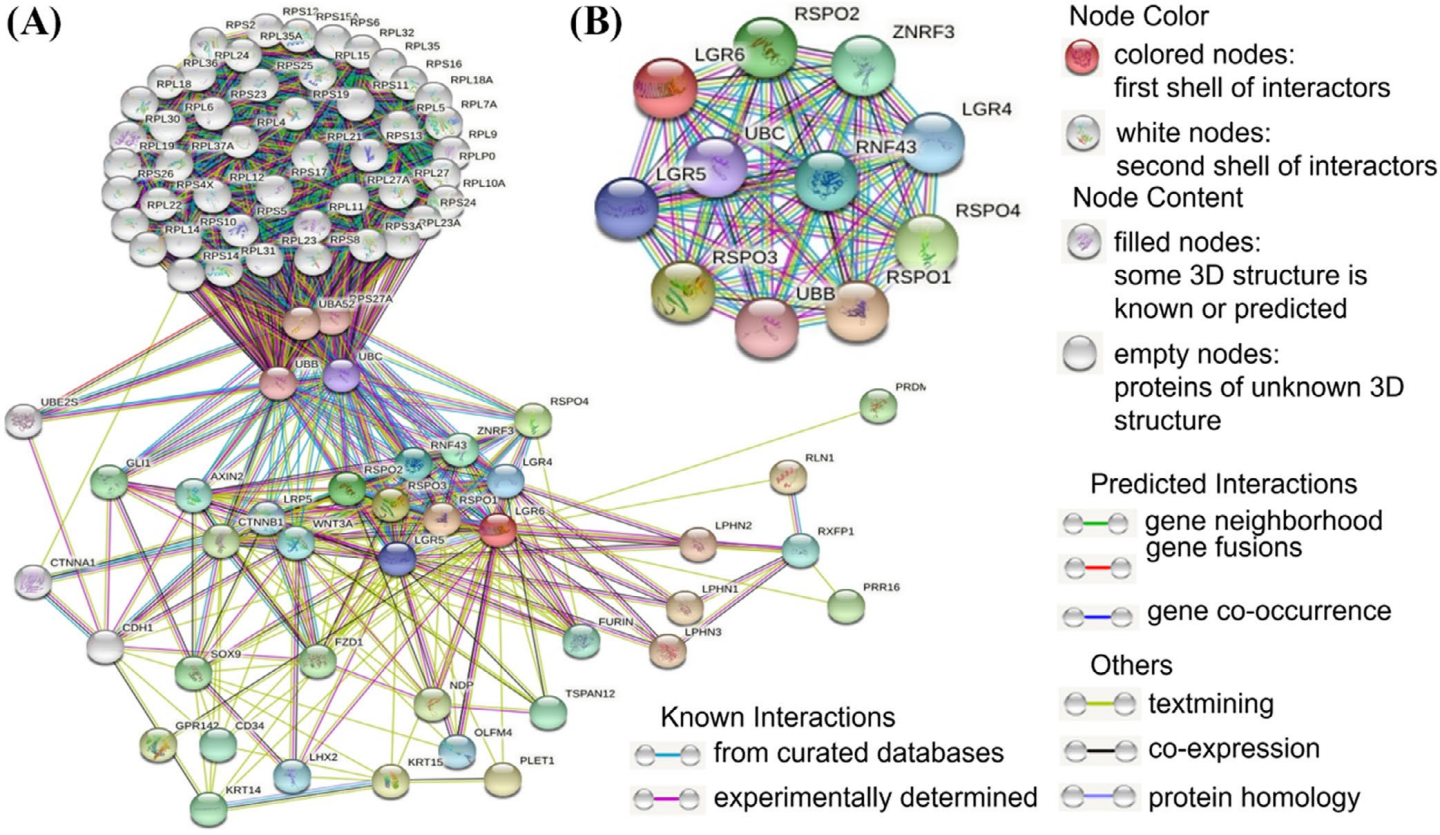
Protein‐protein interaction (PPI) network of LGR6‐related genes. The PPI network was drawn using STRING online. A, Shows 36 nodes interacting with the first shell of LGR6. The minimum required interaction score is 0.41 (moderate confidence). Disconnected nodes are hidden in the network. B, Shows the first ten proteins that interact with LGR6. The minimum required interaction score is 0.905 (very high confidence)

**Figure 7:**
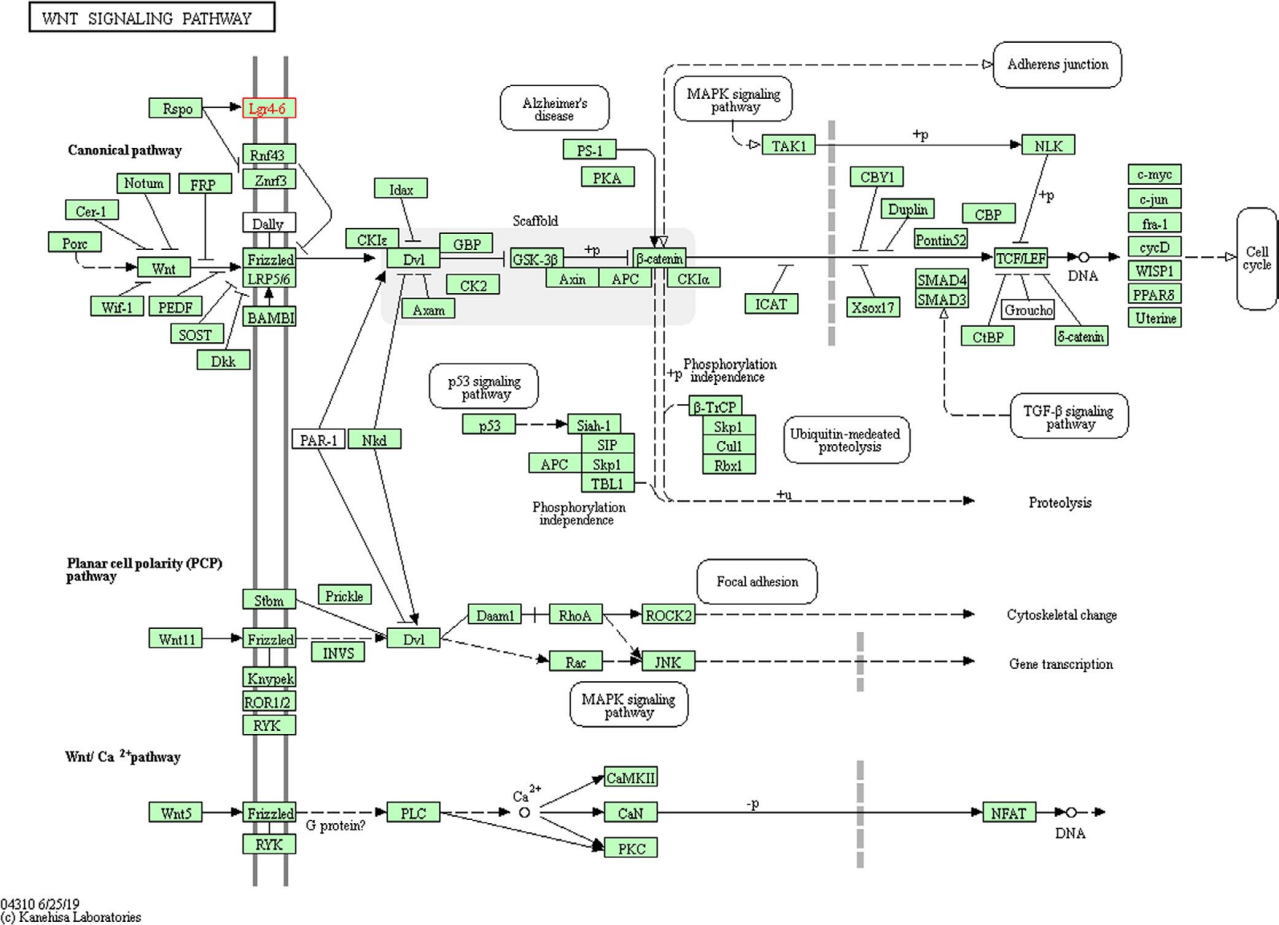
LGR6 KEGG pathway. The KEGG pathway was constructed by the Kyoto Encyclopedia of Genes and Genomes (KEGG) pathway database. The LGR6 KEGG pathway reflects the practical biological functions of LGR6 mediated through the Wnt signalling pathway

PPI networks consist of nodes representing proteins and edges describing related interactions. A PPI network containing 87 nodes and 1528 edges of LGR6‐related genes was established. Figure 6A shows 36 nodes interacting with the first shell of LGR6. The minimum required interaction score was 0.41 (moderate confidence). Figure 6B shows the first ten proteins that interact with LGR6: RSPO1, RSPO2, RSPO3, RSPO4, ZNRF3, RNF43, LGR4, LGR5, UBC, and UBB. The minimum required interaction score was 0.905 (very high confidence). RSPO1, RSPO2, RSPO3, and RSPO4 (interaction score ≧0.967) are activators of the canonical Wnt signaling pathway by acting as a ligand for LGR4‐6 receptors. However, our KEGG pathway analysis also showed that LGR6 functions primarily through its participation in the Wnt/β‐catenin signaling pathway (Figure 7).

## 4 DISCUSSION

The malignancy of esophageal cancer is high, and its prognosis is poor. Predictive markers and effective molecular therapy targets are urgently needed to improve the prognosis of patients with esophageal cancer.

LGR6 has been widely reported to be an important stem cell marker in many cancer types that promotes the occurrence and progression of cancer.^19^, ^20^ Paradoxically, LGR6 has been reported to act as a tumor suppressor gene in colon and breast cancers.^21^, ^22^ These results suggest that the role of LGR6 in tumors is related to the tumor type. The clinical significance and function of LGR6 in ESCC have not been previously reported.

In this study, by accessing the TCGA and UCSC data sets, we found that the expression of LGR6 in normal human esophageal tissues was relatively low at the transcription level, while the expression of LGR6 in esophageal cancer tissues of patients with esophageal cancer was significantly upregulated compared with that in the corresponding surrounding normal tissues. Experimentally, we verified that LGR6 mRNA and protein levels were significantly higher in ESCC tissues than in adjacent normal tissues. We explored the relationship between the expression of LGR6 in cancer tissues from 102 ESCC patients and their clinicopathological features. We found that the upregulation of LGR6 is closely related to a low level of differentiation of ESCC and a poor prognosis of the patients.

Through further analysis of the LGR6 PPI network and KEGG pathways, we found that LGR6 interacts with RSPO1, RSPO2, RSPO3, and RSPO4 (interaction score ≧0.967), and LGR6 primarily participates in the Wnt/β‐catenin signaling pathway to exert its biological functions. LGR6 interacts with its ligand RSPO1‐4 to activate the Wnt/β‐catenin signaling pathway through phosphorylation, thereby affecting multipotent biological functions.^21^, ^23^, ^24^, ^25^

The Wnt/β‐catenin signaling pathway plays a key role in the pathogenesis of various human diseases and tumors.^23^ LGR6 promotes the progression and invasion of lung cancer and high‐grade serous ovarian carcinoma, as well as the stemness and chemoresistance of ovarian cancer cells through this mechanism.^18^, ^19^, ^26^ LGR6 may contribute to the development and progression of ESCC by activating the Wnt/β‐catenin signaling pathway, but further research is needed to confirm this hypothesis.

However, the function of LGR6 in different cancers remains controversial.^19^, ^20^, ^21^, ^22^ Notably, the LGR6 PPI network showed that LGR6 interacts with ZNRF3, RNF43, UBC, UBB and other protein molecules. The minimum required interaction score was 0.905 (very high confidence). ZNRF3 (interaction score: 0.960) and RNF43 (interaction score: 0.960) act as negative regulators of the Wnt signaling pathway and inhibit tumor progression.^23^ UBC and UBB are mainly involved in the ubiquitination of eukaryotic cells, a series of processes including protein degradation, DNA repair, transcription, protein transport, cell cycle regulation, and signal transduction. Many of these processes are crucial for cancer cell survival.^24^ We believe that the ultimate role of LGR6 in the pathogenesis of cancer is the result of the integration of all of its biological functions. Therefore, LGR6 functions differently in different cancers or individuals.

Hauser AS and Lappano R et al reported that G protein–coupled receptors and membrane signaling proteins including LGR6 are good candidates for cancer‐targeting molecular therapies.^25^, ^26^ Our results suggest that LGR6 may be a potential therapeutic target for ESCC, providing a potential therapeutic strategy by increasing the degradation or reducing the expression of LGR6 to interfere with tumor progression.

The limitation of this study is that there was no in‐depth study of the molecular mechanisms of LGR6 in the development and progression of esophageal cancer, which is where further research and exploration are needed in the future.

In summary, this study confirmed for the first time that LGR6 is highly expressed in ESCC tissues and that increased expression of LGR6 is associated with a poor prognosis of ESCC patients. LGR6 may promote ESCC progression by activating the Wnt/β‐catenin signaling pathway. These findings provide a basis for the potential application of LGR6 as a biomarker for early diagnosis and as a target gene for early therapeutic intervention.

## CONFLICTS OF INTEREST

The authors have no conflicts of interest to disclose.
