# Supplementary material for: LGR6 is a potential diagnostic and prognostic marker for esophageal squamous cell carcinoma
Source: J Clin Lab Anal. 2020 Jan 9;34(4):e23121. doi: 10.1002/jcla.23121 (PMC7171331; doi:10.1002/jcla.23121)
Supplement: Supplementary file 1 [file JCLA-34-e23121-s001.docx]

**Supplementary Table 1** Integrated 102 ESCC raw data of two chips

| Number | Age | Gender | Status | Survival time | Clinical stage | T | N | M | Differentiation | IHC score of ESCC | IHC score of NT |
| --- | --- | --- | --- | --- | --- | --- | --- | --- | --- | --- | --- |
| 1 | 47 | female | alive | 60 | Ⅰ | 2 | 0 | 0 | high | 3 | 0 |
| 2 | 48 | male | alive | 60 | Ⅱ | 3 | 0 | 0 | middle | 4 | 1 |
| 3 | 49 | male | dead | 13 | Ⅲ | 4 | 0 | 0 | low | 3 | 1 |
| 4 | 49 | male | dead | 58 | Ⅱ | 2 | 0 | 0 | low | 12 | 0 |
| 5 | 49 | male | alive | 60 | Ⅰ | 1 | 0 | 0 | high | 3 | 3 |
| 6 | 50 | male | alive | 60 | Ⅲ | 4 | 2 | 0 | high | 6 | 2 |
| 7 | 51 | male | dead | 49 | Ⅱ | 3 | 0 | 0 | low | 12 | 3 |
| 8 | 52 | female | dead | 41 | Ⅲ | 2 | 1 | 0 | high | 2 | 0 |
| 9 | 52 | female | alive | 60 | Ⅱ | 3 | 0 | 0 | high | 3 | 0 |
| 10 | 53 | female | dead | 31 | Ⅲ | 4 | 1 | 0 | high | 3 | 1 |
| 11 | 54 | male | dead | 39 | Ⅲ | 3 | 3 | 0 | low | 3 | 2 |
| 12 | 54 | female | dead | 44 | Ⅱ | 3 | 0 | 0 | high | 9 | 2 |
| 13 | 54 | male | alive | 60 | Ⅲ | 3 | 1 | 0 | high | 3 | 1 |
| 14 | 55 | female | alive | 60 | Ⅱ | 3 | 0 | 0 | low | 12 | 4 |
| 15 | 55 | female | alive | 60 | Ⅰ | 3 | 0 | 0 | high | 4 | 6 |
| 16 | 55 | male | alive | 60 | Ⅰ | 2 | 0 | 0 | low | 9 | 0 |
| 17 | 56 | female | dead | 15 | Ⅲ | 3 | 1 | 0 | low | 9 | 2 |
| 18 | 56 | male | dead | 35 | Ⅱ | 3 | 0 | 0 | low | 12 | 0 |
| 19 | 56 | male | dead | 40 | Ⅲ | 3 | 3 | 0 | high | 3 | 1 |
| 20 | 56 | male | alive | 60 | Ⅰ | 1 | 0 | 0 | middle | 3 | 2 |
| 21 | 57 | male | dead | 9 | Ⅱ | 3 | 0 | 0 | middle | 9 | 0 |
| 22 | 57 | female | dead | 13 | Ⅱ | 3 | 0 | 0 | middle | 9 | 12 |
| 23 | 57 | male | alive | 60 | Ⅲ | 3 | 2 | 0 | high | 2 | 0 |
| 24 | 58 | female | dead | 4 | Ⅲ | 2 | 1 | 0 | middle | 8 | 2 |
| 25 | 58 | female | dead | 15 | Ⅲ | 3 | 2 | 0 | low | 9 | 0 |
| 26 | 58 | female | dead | 37 | Ⅲ | 3 | 1 | 0 | middle | 9 | 2 |
| 27 | 58 | male | alive | 60 | Ⅱ | 3 | 0 | 0 | middle | 3 | 0 |
| 28 | 58 | male | alive | 60 | Ⅰ | 1 | 0 | 0 | high | 1 | 0 |
| 29 | 59 | female | dead | 12 | Ⅲ | 4 | 2 | 0 | low | 12 | 0 |
| 30 | 59 | male | dead | 17 | Ⅲ | 3 | 2 | 0 | middle | 9 | 0 |
| 31 | 59 | male | dead | 19 | Ⅰ | 1 | 0 | 0 | high | 8 | 2 |
| 32 | 59 | female | dead | 57 | Ⅲ | 3 | 2 | 0 | high | 3 | 1 |
| 33 | 59 | female | alive | 60 | Ⅰ | 1 | 0 | 0 | high | 0 | 0 |
| 34 | 60 | male | dead | 14 | Ⅰ | 2 | 0 | 0 | low | 12 | 0 |
| 35 | 60 | male | dead | 24 | Ⅲ | 2 | 2 | 0 | low | 9 | 2 |
| 36 | 60 | male | alive | 60 | Ⅰ | 1 | 0 | 0 | low | 12 | 0 |
| 37 | 61 | male | dead | 10 | Ⅲ | 3 | 3 | 0 | low | 9 | 0 |
| 38 | 61 | male | dead | 12 | Ⅲ | 3 | 1 | 0 | low | 12 | 2 |
| 39 | 61 | male | dead | 20 | Ⅲ | 3 | 1 | 0 | middle | 8 | 2 |
| 40 | 61 | male | dead | 36 | Ⅱ | 3 | 0 | 0 | middle | 6 | 1 |
| 41 | 61 | male | alive | 60 | Ⅱ | 3 | 0 | 0 | high | 3 | 4 |
| 42 | 62 | female | dead | 8 | Ⅲ | 1 | 3 | 0 | low | 9 | 2 |
| 43 | 62 | female | dead | 40 | Ⅱ | 3 | 0 | 0 | middle | 12 | 0 |
| 44 | 62 | female | dead | 49 | Ⅲ | 3 | 1 | 0 | high | 3 | 2 |
| 45 | 63 | male | dead | 21 | Ⅱ | 2 | 0 | 0 | high | 9 | 2 |
| 46 | 63 | female | dead | 29 | Ⅱ | 3 | 0 | 0 | middle | 6 | 0 |
| 47 | 63 | male | dead | 47 | Ⅲ | 3 | 2 | 0 | high | 4 | 2 |
| 48 | 63 | male | alive | 60 | Ⅰ | 1 | 0 | 0 | high | 3 | 0 |
| 49 | 64 | male | dead | 36 | Ⅲ | 4 | 0 | 0 | high | 3 | 0 |
| 50 | 64 | male | dead | 57 | Ⅲ | 3 | 3 | 0 | low | 3 | 1 |
| 51 | 64 | male | alive | 60 | Ⅱ | 2 | 0 | 0 | middle | 8 | 0 |
| 52 | 64 | male | alive | 60 | Ⅰ | 3 | 0 | 0 | high | 3 | 0 |
| 53 | 64 | female | alive | 60 | Ⅱ | 2 | 0 | 0 | high | 3 | 4 |
| 54 | 65 | female | dead | 18 | Ⅱ | 1 | 1 | 0 | middle | 8 | 0 |
| 55 | 65 | male | dead | 19 | Ⅲ | 3 | 1 | 0 | low | 12 | 1 |
| 56 | 65 | female | dead | 22 | Ⅲ | 3 | 1 | 0 | middle | 8 | 2 |
| 57 | 65 | male | alive | 60 | Ⅲ | 3 | 1 | 0 | middle | 9 | 1 |
| 58 | 65 | male | alive | 60 | Ⅲ | 3 | 1 | 0 | middle | 2 | 0 |
| 59 | 65 | female | alive | 60 | Ⅰ | 2 | 0 | 0 | high | 3 | 0 |
| 60 | 66 | male | dead | 13 | Ⅲ | 3 | 1 | 0 | high | 8 | 2 |
| 61 | 66 | female | dead | 20 | Ⅱ | 1 | 1 | 0 | middle | 8 | 0 |
| 62 | 66 | male | dead | 43 | Ⅱ | 2 | 1 | 0 | low | 9 | 0 |
| 63 | 67 | male | dead | 10 | Ⅱ | 3 | 0 | 0 | middle | 9 | 3 |
| 64 | 67 | female | alive | 60 | Ⅱ | 3 | 0 | 0 | high | 6 | 0 |
| 65 | 67 | female | alive | 60 | Ⅱ | 3 | 0 | 0 | high | 9 | 1 |
| 66 | 68 | female | alive | 60 | Ⅱ | 3 | 0 | 0 | middle | 6 | 0 |
| 67 | 68 | female | alive | 60 | Ⅰ | 1 | 0 | 0 | high | 2 | 0 |
| 68 | 69 | female | alive | 60 | Ⅱ | 3 | 0 | 0 | high | 1 | 3 |
| 69 | 69 | female | alive | 60 | Ⅱ | 3 | 0 | 0 | high | 8 | 2 |
| 70 | 69 | male | alive | 60 | Ⅱ | 3 | 0 | 0 | high | 3 | 1 |
| 71 | 70 | male | dead | 11 | Ⅲ | 3 | 2 | 0 | low | 9 | 0 |
| 72 | 70 | male | dead | 22 | Ⅲ | 3 | 2 | 0 | high | 6 | 1 |
| 73 | 70 | male | dead | 25 | Ⅲ | 3 | 3 | 0 | middle | 1 | 0 |
| 74 | 70 | male | dead | 50 | Ⅱ | 3 | 0 | 0 | middle | 3 | 3 |
| 75 | 70 | male | alive | 60 | Ⅰ | 1 | 0 | 0 | middle | 0 | 0 |
| 76 | 70 | male | alive | 60 | Ⅱ | 2 | 0 | 0 | middle | 9 | 2 |
| 77 | 70 | male | alive | 60 | Ⅱ | 2 | 0 | 0 | high | 0 | 0 |
| 78 | 71 | male | dead | 11 | Ⅲ | 3 | 3 | 0 | low | 8 | 2 |
| 79 | 71 | female | dead | 14 | Ⅰ | 1 | 0 | 0 | middle | 9 | 2 |
| 80 | 71 | male | dead | 16 | Ⅱ | 3 | 0 | 0 | middle | 12 | 0 |
| 81 | 71 | female | dead | 33 | Ⅰ | 1 | 0 | 0 | high | 6 | 1 |
| 82 | 71 | male | dead | 37 | Ⅱ | 3 | 0 | 0 | middle | 6 | 1 |
| 83 | 71 | female | dead | 38 | Ⅲ | 4 | 1 | 0 | high | 6 | 2 |
| 84 | 71 | male | dead | 53 | Ⅱ | 2 | 1 | 0 | middle | 3 | 2 |
| 85 | 71 | male | alive | 60 | Ⅲ | 3 | 1 | 0 | middle | 3 | 0 |
| 86 | 72 | male | dead | 13 | Ⅱ | 3 | 0 | 0 | middle | 12 | 0 |
| 87 | 72 | male | alive | 60 | Ⅲ | 4 | 1 | 0 | middle | 9 | 1 |
| 88 | 73 | female | dead | 12 | Ⅱ | 2 | 0 | 0 | middle | 12 | 0 |
| 89 | 73 | male | dead | 20 | Ⅱ | 2 | 1 | 0 | middle | 2 | 0 |
| 90 | 73 | male | dead | 45 | Ⅲ | 3 | 2 | 0 | high | 1 | 0 |
| 91 | 73 | male | alive | 60 | Ⅱ | 3 | 0 | 0 | middle | 3 | 1 |
| 92 | 74 | female | dead | 34 | Ⅲ | 3 | 1 | 0 | high | 4 | 2 |
| 93 | 74 | male | alive | 60 | Ⅲ | 3 | 1 | 0 | high | 2 | 1 |
| 94 | 75 | female | alive | 60 | Ⅲ | 3 | 1 | 0 | high | 4 | 1 |
| 95 | 75 | female | alive | 60 | Ⅲ | 3 | 1 | 0 | high | 2 | 0 |
| 96 | 76 | female | dead | 12 | Ⅲ | 2 | 1 | 0 | middle | 8 | 2 |
| 97 | 77 | female | dead | 14 | Ⅱ | 3 | 0 | 0 | middle | 8 | 0 |
| 98 | 77 | female | dead | 30 | Ⅱ | 2 | 0 | 0 | low | 12 | 0 |
| 99 | 77 | male | alive | 60 | Ⅰ | 2 | 0 | 0 | middle | 6 | 1 |
| 100 | 78 | male | dead | 12 | Ⅱ | 3 | 0 | 0 | middle | 9 | 0 |
| 101 | 78 | female | dead | 56 | Ⅲ | 3 | 1 | 0 | high | 1 | 0 |
| 102 | 79 | male | dead | 53 | Ⅲ | 1 | 2 | 0 | middle | 4 | 2 |
